# **Contact H**ypersensitivity to Oxazolone Provokes Vulvar Mechanical Hyperalgesia in Mice

**DOI:** 10.1371/journal.pone.0078673

**Published:** 2013-10-25

**Authors:** Tijana Martinov, Rose Glenn-Finer, Sarah Burley, Elena Tonc, Evelyn Balsells, Alyssa Ashbaugh, Linnea Swanson, Randy S. Daughters, Devavani Chatterjea

**Affiliations:** 1 Department of Biology, Macalester College, St. Paul, Minnesota, United States of America; 2 Stem Cell Institute, University of Minnesota, Minneapolis, Minnesota, United States of America; Northwestern University, United States of America

## Abstract

The interplay among pain, allergy and dysregulated inflammation promises to yield significant conceptual advances in immunology and chronic pain. Hapten-mediated contact hypersensitivity reactions are used to model skin allergies in rodents but have not been utilized to study associated changes in pain perception in the affected skin. Here we characterized changes in mechanical hyperalgesia in oxazolone-sensitized female mice challenged with single and repeated labiar skin exposure to oxazolone. Female mice were sensitized with topical oxazolone on their flanks and challenged 1-3 times on the labia. We then measured mechanical sensitivity of the vulvar region with an electronic pressure meter and evaluated expression of inflammatory genes, leukocyte influx and levels of innervation in the labiar tissue. Oxazolone-sensitized mice developed vulvar mechanical hyperalgesia after a single labiar oxazolone challenge. Hyperalgesia lasted up to 24 hours along with local influx of neutrophils, upregulation of inflammatory cytokine gene expression, and increased density of cutaneous labiar nerve fibers. Three daily oxazolone challenges produced vulvar mechanical hyperalgesic responses and increases in nerve density that were detectable up to 5 days post-challenge even after overt inflammation resolved. This persistent vulvar hyperalgesia is resonant with vulvodynia, an understudied chronic pain condition that is remarkably prevalent in 18-60 year-old women. An elevated risk for vulvodynia has been associated with a history of environmental allergies. Our pre-clinical model can be readily adapted to regimens of chronic exposures and long-term assessment of vulvar pain with and without concurrent inflammation to improve our understanding of mechanisms underlying subsets of vulvodynia and to develop new therapeutics for this condition.

## Introduction

Rodent models of contact hypersensitivity (CHS) help elucidate the underlying mechanisms of skin allergies such as atopic dermatitis [1,2]. The CHS response to the hapten oxazolone (Ox) depends on mast cell immunoglobulin (Ig) E [3,4], and is accompanied by tissue edema [3,5], upregulation of inflammatory cytokines [4,6,7] and mast cell-dependent cutaneous nerve elongation [5]. However, mechanistic connections between allergic reactions and subsequent changes in pain perception in the skin remain largely understudied. Provoked, burning vulvar pain, or vulvodynia [8] has been associated with a history of seasonal allergies [9]. Women with vulvodynia show increased mast cell numbers in vulvar biopsies [10] and hyper-innervation of the vulvo-vestibular tissue [11,12] suggesting that underlying pathological mechanisms may be similar to those reported in rodent models of Ox-CHS [5]. 

Here we demonstrate that in Ox-sensitized female ND4 Swiss mice, a single labiar Ox challenge provokes vulvar mechanical hyperalgesia for up to 24 hours and is accompanied by neutrophil influx, upregulation of inflammatory cytokine gene expression, and increased density of cutaneous calcitonin gene related peptide (CGRP)^+^ and protein gene product 9.5 (PGP 9.5)^+^ nerve fibers. Following 3 daily consecutive Ox challenges, vulvar hyperalgesia persists for up to 5 days, after the tissue markers of overt inflammation have resolved, demonstrating long-term changes in mechanical sensitivity in the affected skin. 

We provide the first evidence of induction of measurable CHS-associated hyperalgesia in mice and establish the first pre-clinical model of allergen-provoked vulvar pain. The model we present here can be adapted to chronic labiar allergen exposures and long-term assessment of pain with and without ongoing inflammation to dissect the complex interplay between allergies and chronic pain in vulvodynia and beyond.

## Materials and Methods

### Ethics statement and animal usage

8-12 week old female ND4 Swiss mice (Harlan Laboratories, Indianapolis, IN) were housed in Macalester College’s animal facility in accordance with National Institutes of Health-approved guidelines, with a 12-hour light/dark cycle and free access to food and water, and were used with Institutional Animal Care and Use Committee approval (Macalester College IACUC protocols B11F1 and B13S1) for these studies.

### Drug administration

Mice were sensitized with topical application of 2% Ox (100 µl; Sigma-Aldrich, St. Louis, MO) on their shaved flanks on day 1, and challenged on the shaved labia with 1% Ox (40 µl) or vehicle (100% ethanol; EtOH) on day 5 [5] or on days 5-7 [13] (Figure S1). Treatments were performed at the same time of day as circadian regulation can affect CHS [14]. 

### Mechanical hyperalgesia assessment

To measure vulvar sensitivity, mice were stimulated along the ano-genital ridge with a semi-flexible polypropylene tip fitted to an electronic von Frey pressure meter (IITC Life Science, Woodland Hills, CA) until they demonstrated one or more nociceptive behaviors: genital licking, lower abdomen retraction, a back paw jump, or a four paw jump [15]. Baseline measurements were taken 24 and 48 hours before sensitization; of 5 baseline measurements per mouse, three closest to the median were averaged to obtain the baseline withdrawal threshold as previously described [16]. Mice with baseline withdrawal threshold average lower than 0.50 grams and mice with baseline thresholds differing by more than 1.00 gram were excluded from the experiment. Remaining mice were assigned to treatment groups such that average baseline thresholds across treatment groups were as similar as possible as previously described [16]. Experimental withdrawal threshold was calculated as the average of 3-4 measurements taken at stated time points after Ox challenge [17]. Delta withdrawal threshold for each mouse was obtained by subtracting its own average baseline from its own average experimental threshold. At least 10 mice were used per treatment group for each experiment and data shown represent 2-3 independent experiments. After hyperalgesia measurements were performed, mice were euthanized and 3-6 mice per treatment group from each experiment were used for the analyses described below.

### Quantification of vulvar myeloperoxidase activity

Labia were preserved and processed as described [17], and absorbance measured at 450 nm to measure myeloperoxidase activity (normalized to tissue weight, and represented as optical density i.e. OD/g of wet tissue).

### RNA isolation and quantitative real-time RT-PCR (qRT-PCR)

Total RNA was extracted from flash-frozen labiar tissue (Total RNA Mini Kit, Midwest Scientific, St. Louis, MO), quantified with Nanodrop ND-1000 Spectrophotometer (ThermoScientific, Wilmington, DE), and reverse-transcribed in a 2720 Thermal Cycler (Life Technologies, Grand Island, NY) using Superscript III First-Strand Synthesis System (Life Technologies) with 100ng of RNA per reaction. Transcripts were quantified using TaqMan Gene Expression Assay Primer/Probe Sets and TaqMan Master Mix (Life Technologies) in a Bio-Rad iCycler (Bio-Rad, Hercules, CA) using *β-2-microglobulin* (*β2m*; Mm00437762_m1), *interleukin-1β* (*IL-1β*; Mm00434228_m1), *tumor necrosis factor-α* (*TNF-*α; Mm00443260_g1), interleukin-6 (IL-6; Mm00446190_m1), *chemokine* (*C-X-C motif*) *ligand 2* (*CXCL2*; Mm00436450_m1), *chemokine* (*C-X-C motif*) *ligand 1* (*CXCL1*; Mm04207460_m1), and *interferon-* γ (*IFN-γ*; Mm01168134_m1) primer/probe sets, normalized to *β2m*, and calculated as fold-expression over controls [18]. 

### Immuno-fluorescent staining and analysis

Labiar tissue samples embedded in Optimal Cutting Temperature compound (Sakura Finetek, Torrance, CA) and snap frozen over liquid nitrogen were cryo-sectioned (16-20 µm), post-fixed in 2% paraformaldehyde (Sigma-Aldrich; pH 8.5) for 5 minutes (room temperature), permeabilized with 0.1% Triton/PBS for 10 minutes, blocked with 5% normal goat serum/PBS, incubated overnight (4°C) with 1:400 anti-CGRP (Abbiotec, San Diego, CA) and then with Alexa Fluor 488-conjugated secondary antibody (Life Technologies; 1:1000) in blocking solution for 2 hours (room temperature). Slides were cover-slipped with Vectashield + DAPI (Vector Laboratories, Burlingame, CA). We performed a quantitative assessment of nerve density on images obtained from confocal sections and analyzed using Fluoview 1000 image analysis software (Olympus Corporation, Center Valley, PA). Nerve density values were determined from randomly chosen regions of interest within tissue sections and were defined as the fluorescent pixel intensity of CGRP or PGP9.5 labeled nerves within a 5mm^2^ area. Average density values per sample (n=3 per treatment group) were calculated from four regions of interest per section for five sections per sample. Each image analyzed was a composite of fifteen optical sections projected along the z-axis in 1µm increments.

### Statistical analysis

Data were processed with Excel (Microsoft, Redmond, WA), graphed with PRISM 5.0 (GraphPad, San Diego, CA) and shown as mean ± SEM. One-way ANOVA and Tukey HSD *post hoc* analysis were performed using JMP Software (v. 9.0) (SAS, Cary, NC). Statistical significance was defined as *p*<0.05. 

## Results

### Single vulvar Ox challenge caused allergic vulvar hyperalgesia, neutrophil influx and upregulation of inflammatory gene expression in Ox-sensitized female ND4 Swiss mice

**Figure 1:**
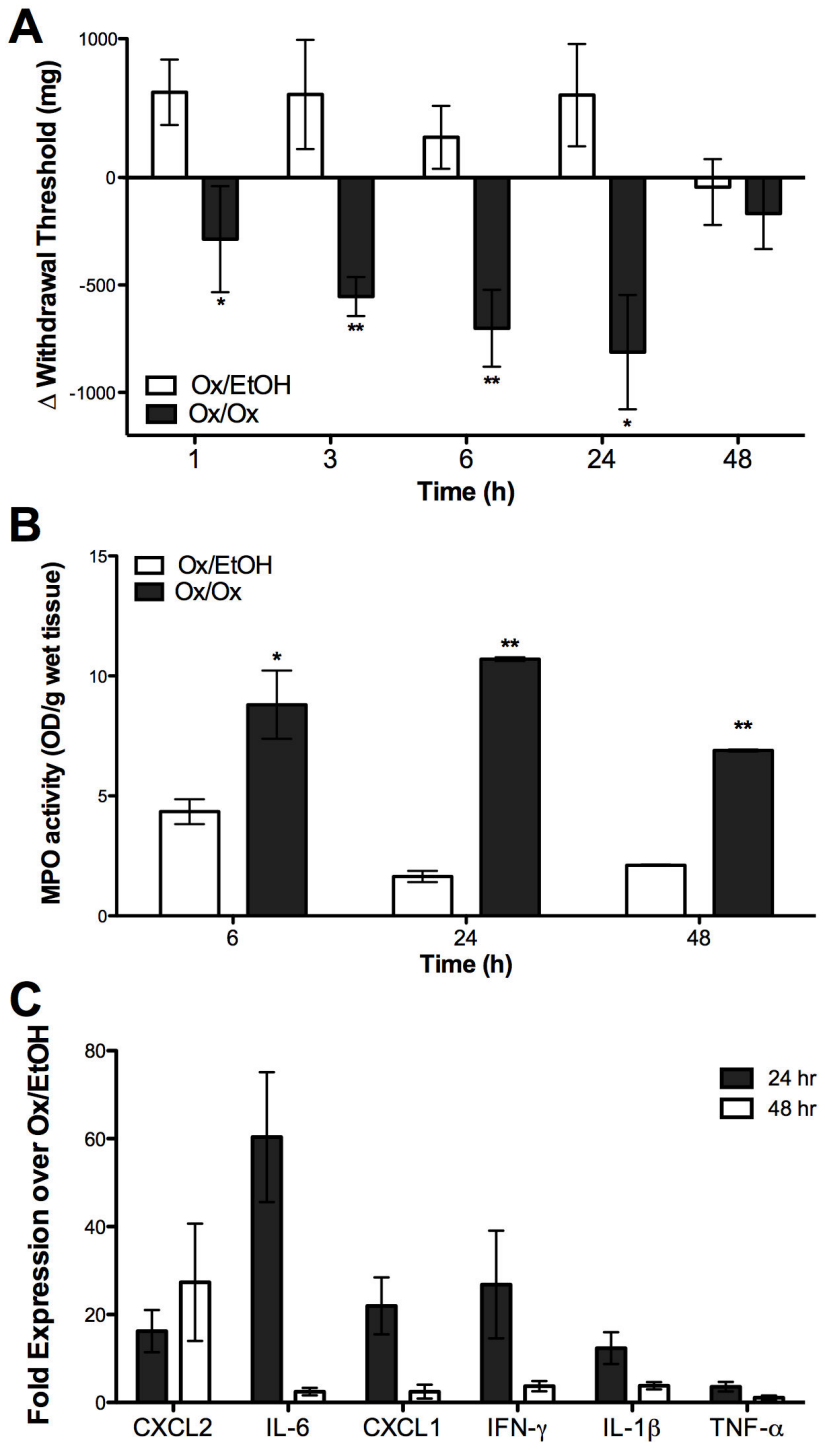
Labiar oxazolone challenge induces acute vulvar mechanical hyperalgesia in sensitized female ND4 Swiss mice. Mice that received a single oxazolone challenge following sensitization show increased mechanical hyperalgesia (A), increased myeloperoxidase activity in the labiar tissue (B), and increased abundance of *CXCL2*, *IL-6*, *CXCL-1*, *IFN-γ*, *IL-1β*, and *TNF-α*mRNAs (C). Significances are compared to Ox/EtOH (* = p<0.05, ** = p<0.01, *** = p< 0.0001respectively). n=3-10 mice per treatment group; data represent at least 2-3 independent experiments.

Oxazolone-sensitized ND4 Swiss mice were significantly more sensitive to vulvar mechanical pressure measured at 1, 3, 6, and 24 hours after when challenged on day 4 following sensitization with 1% Ox on the labia compared to vehicle-challenged controls; hyperalgesia peaked at 24 hours and resolved by 48 hours (Figure 1A). Farmer and colleagues [15] have previously defined hyperalgesic mice as those that have 33% or greater reduction in experimental vulvar withdrawal thresholds compared to baseline. Using this criterion, we found that following a single Ox challenge, 67-100% of mice were hyperalgesic compared to 0-12.5% of vehicle-challenged mice over the time points at which mechanical sensitivity was significantly different between treatment groups (Table 1). Neither the mice’s stage of estrous nor Ox sensitization alone produced hyperalgesic responses (Figure S2A-B). Sensitivity was restricted to the Ox challenge site and could not be detected in the hind paw (Figure S2C).

**Table 1:** Percentages of mice showing hyperalgesic vulvar responses (i.e. vulvar withdrawal thresholds ≤ 33% of baseline) following single, and triple, labiar Ox or vehicle challenge.

**Time (h)**	**1**	**3**	**6**	**24**	**48**	**120**	**240**
**Ox/EtOH (1)**	0	12.5	0	0	28.5	-	-
**Ox/Ox (1)**	66.67	100	88.9	83.3	35.7	-	-
**Ox/EtOH (3)**	-	-	34.80	40.90	-	57.10	44.40
**Ox/Ox (3)**	-	-	70.40	65.40	-	82.60	62.50

We next examined leukocyte infiltration and inflammatory cytokine gene expression in the labia. Vulvar myeloperoxidase activity indicating infiltrating neutrophils [17] was higher in homogenates from Ox- vs. vehicle-challenged mice at 6, 24, and 48 hours, peaking at 24 hours (Figure 1B). Transcripts encoding cytokines that mediate inflammatory hyperalgesia in rodents [19–21] were markedly upregulated - *CXCL2* (~20-fold), *IL-6* (~60-fold), *CXCL1* (~25-fold), *IFN-γ*(~25-fold), *IL-1β* (~18-fold) and *TNF*-α (~5-fold) - in Ox-challenged vs. vehicle-treated controls at 24 hours but largely resolved by 48 hours after challenge, except for *CXCL2* expression which was variable but remained at around 20-30 fold over ethanol-challenged controls (Figure 1C). IL-1β and TNF-α are some of the earliest cytokines expressed following skin inflammation [22]; here we found that *IL-1β* transcripts were markedly elevated ~60-fold in Ox challenged mice vs. controls as early as 6 hours after a single challenge but there was only a slight increase (<5-fold) in *TNF-α* mRNA levels compared to controls at this early time point (Figure S3).

In sum, Ox-provoked vulvar CHS caused pronounced mechanical hyperalgesia accompanied by neutrophil influx and increase in inflammatory cytokine gene expression. 

### Repeated Ox challenge caused persistent vulvar hyperalgesia in sensitized female ND4 Swiss mice after resolution of overt tissue inflammation

**Figure 2:**
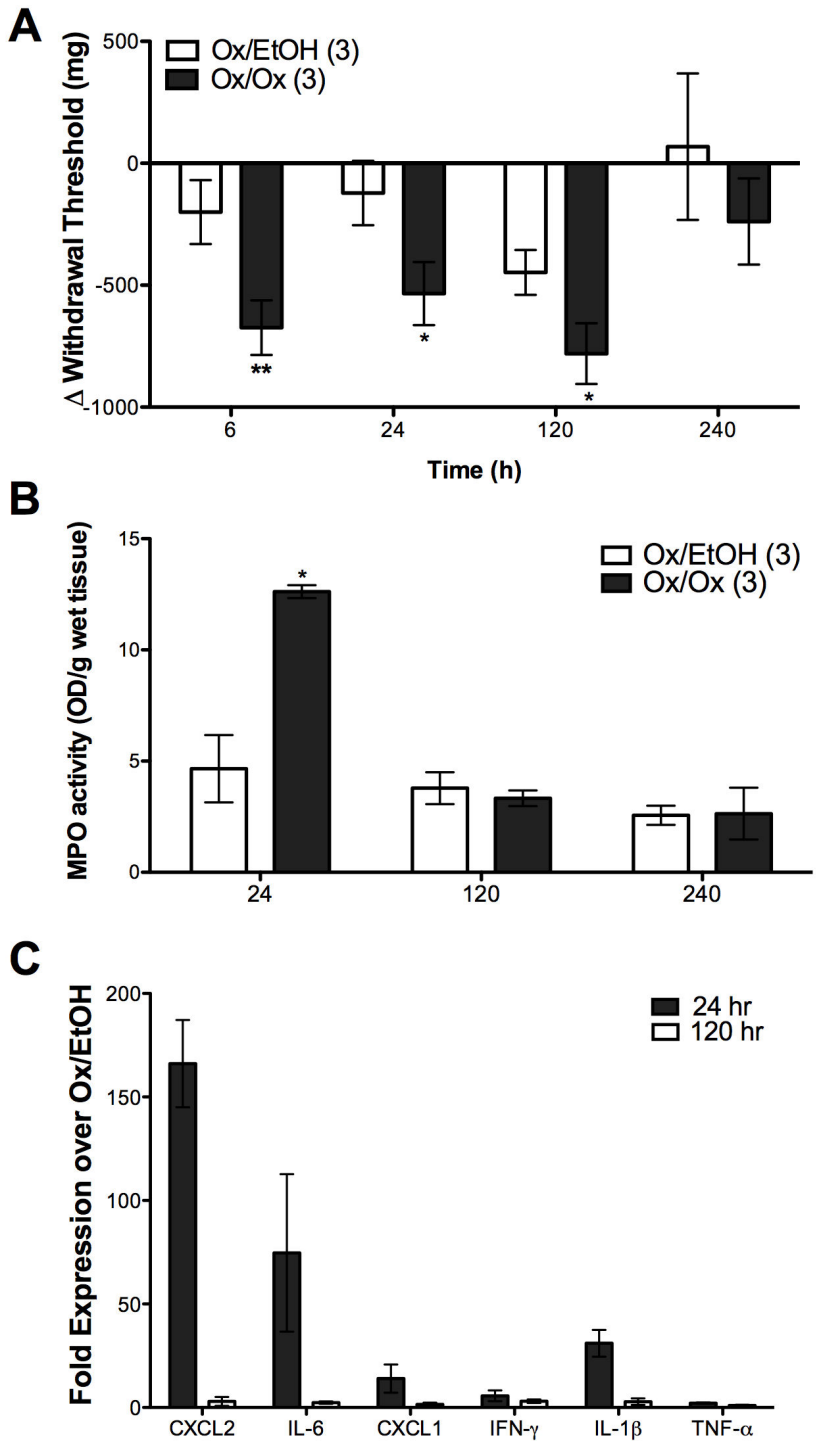
Repeated (3x) labiar oxazolone challenge induces persistent vulvar mechanical hyperalgesia in female ND4 Swiss mice. Ox-sensitized mice given three consecutive labiar challenges show increased mechanical hyperalgesia at 24 hours and 120 hours (5 days) (A), an increase in myeloperoxidase activity at 24 hours (B), an upregulation in proinflammatory cytokine transcript levels (C). Significances are compared to Ox/EtOH (* = p<0.05, ** = p<0.01 respectively). n=3 (myeloperoxidase assays) or n=20-24 (hyperalgesia assays) mice per treatment group; data represent at least 2-3 independent experiments.

We investigated whether repeated Ox challenges in the skin produced longer-lasting vulvar hyperalgesic responses compared to a single challenge. We found that 3 daily labiar challenges with Ox produced expected vulvar mechanical sensitivity at 6 and 24 hours after the third challenge. These responses then persisted for up to 5 days (120 hours) and resolved by 10 days (240 hours) post-challenge (Figure 2A). Of mice challenged thrice with Ox, 70-83% were hyperalgesic (experimental latencies ≤ 33% of their baseline responses) over the time points at which mechanical sensitivities were significantly different between treatment groups (Table 1). Changes in vulvar sensitivity remained localized to site of challenge (data not shown). 

At 24 hours post-challenge, tissue myeloperoxidase levels increased > 2-fold in Ox triple-challenged mice vs. controls. By 5 days (120 hours) after the third challenge, there were no differences in myeloperoxidase activity levels between experimental and control mice (Figure 2B). *CXCL2* transcripts increased ~160-fold over controls at 24h after 3 consecutive Ox challenges but were reduced to <10-fold by 5 days; 24 hours after the third challenge, *IL-6* transcripts were ~ 75-fold more abundant over controls, IL-1β ~ 30-fold elevated over controls. CXCL1 was 15-fold higher than controls, while *IFN-γ* and *TNF-α* mRNAs showed little to no increase (Figure 2C). Similar to our observations following a single Ox challenge, we found that *IL-1β* transcripts were upregulated ~ 12-fold in Ox challenged mice vs. controls as early as 6 hours after the third challenge but there were no changes in *TNF-α* mRNA levels compared to controls at this early time point (Figure S3). As multiple Ox challenges can induce a Th2 cytokine profile [23], we assessed interleukin-4 (IL-4) gene expression 24 hours after 3 Ox challenges. We observed a very slight increase in triple-challenged mice but detected no *IL-4* transcription in untreated and vehicle-challenged controls (Table S1). 

Therefore, vulvar mechanical hyperalgesia persisted for 5 days following 3 daily challenges with Ox in sensitized mice after increases in neutrophil influx and inflammatory cytokine gene expression had resolved to control levels.

### Density of CGRP^+^ and PGP 9.5^+^ cutaneous nerves in the labiar skin of Ox-sensitized female ND4 Swiss mice increased after single and repeated Ox challenges

**Figure 3:**
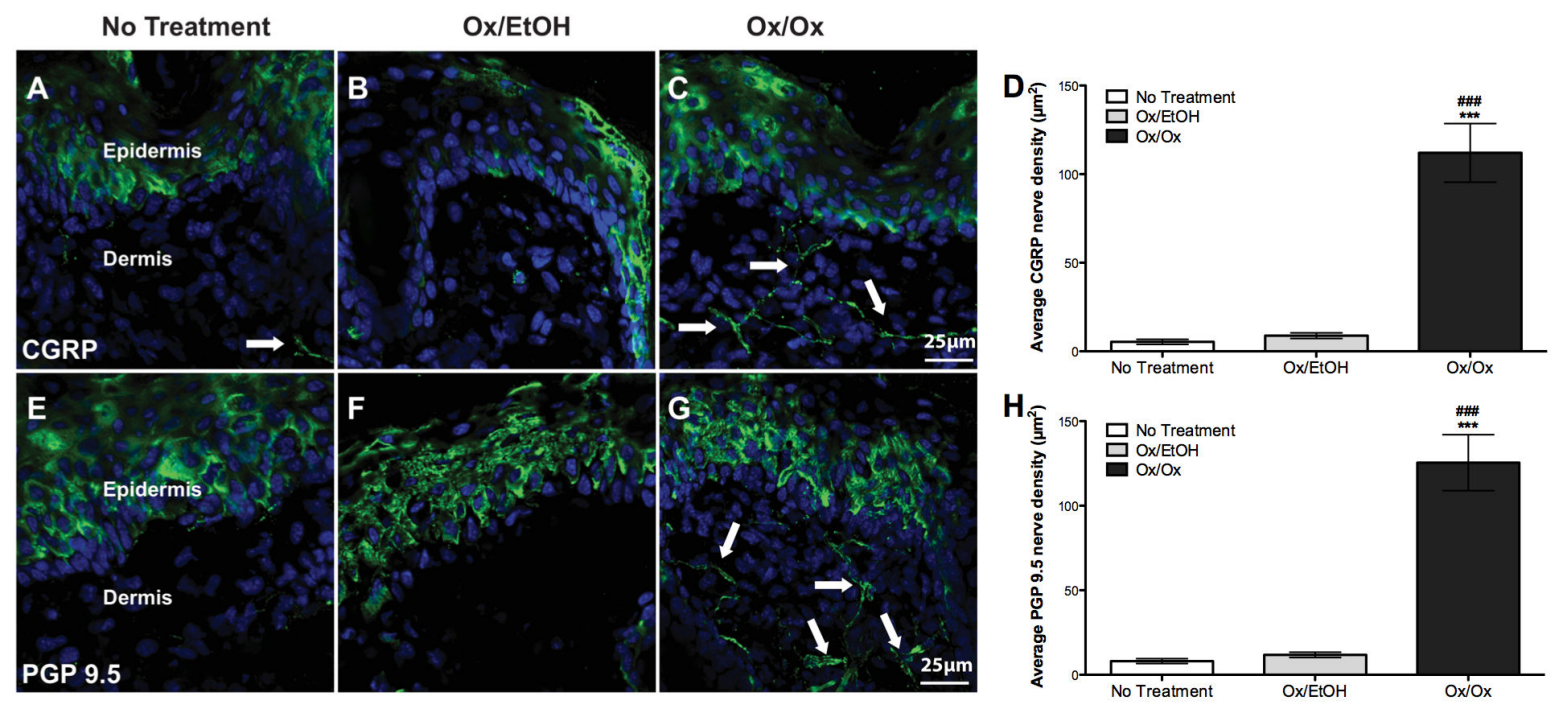
Labiar CGRP^+^ and PGP 9.5^+^ nerve density increases following single oxazolone challenge in sensitized mice. Ox-challenged sensitized mice show ~8-10-fold increased density of cutaneous CGRP^+^(A-D) and PGP 9.5^+^ (E-H) nerves at 48 hours after challenge. Significances are compared to Ox/EtOH (*** = p< 0.0001); and untreated controls (### = p< 0.0001). n=3 mice per treatment group.

**Figure 4:**
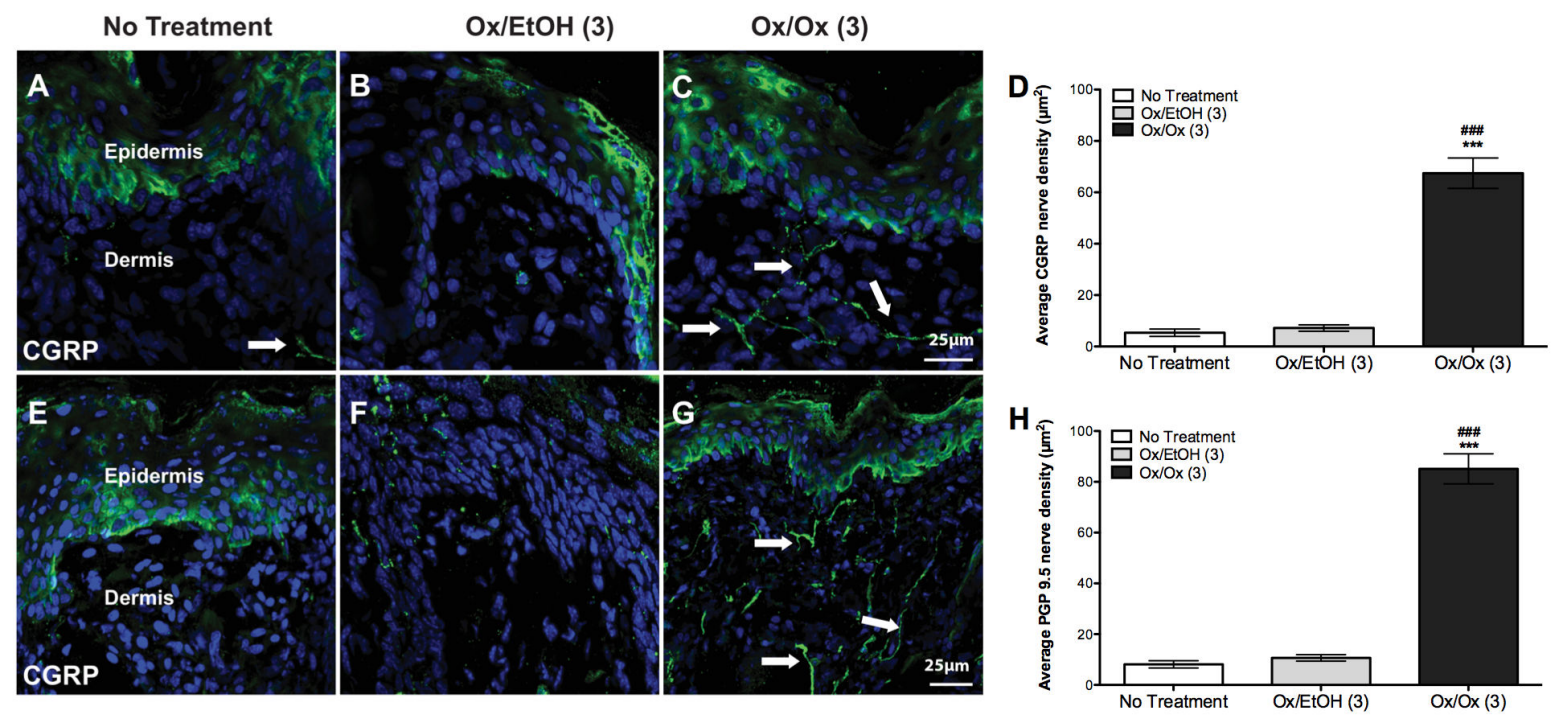
Labiar CGRP^+^ and PGP 9.5^+^ nerve density increases after three oxazolone challenges in sensitized mice. Mice challenged thrice with Ox show ~6-8-fold increased CGRP^+^ (A-D) and PGP 9.5^+^ (E-H) cutaneous nerve density 5 days after 3 consecutive daily Ox challenges. Significances are compared to Ox/EtOH (*** = p< 0.0001); and untreated controls (### = p< 0.0001). n=3 mice per treatment group.

Increased cutaneous innervation has been reported in mouse models of Ox-mediated CHS [5] and in human atopic dermatitis [24]. We found 8-10-fold increase in densities of CGRP^+^ (Figure 3A-C, D) and PGP 9.5^+^ (Figure 3E-G, H) nerve fibers in sensitized mice challenged once with Ox vs. vehicle 48 hours after challenge. Densities of CGRP^+^ (Figure 4A-C, D) and PGP 9.5^+^ (Figure 4E-G, H) cutaneous nerve fibers in the labiar skin 5 days after 3 daily Ox challenges were 6-8 fold greater in Ox triple-challenged skin vs. untreated and vehicle-challenged controls. CGRP labels a subset of nociceptive neurons in contrast to the pan-neuronal marker PGP 9.5 [5]; accordingly, we visually observed more cutaneous staining with the latter vs. the former marker. However, the fold increases in cutaneous density seen after 1 or 3 Ox challenges were comparable for both neuronal markers with a slightly smaller increase observed at 120 hours after 3 challenges compared to 48 hours after a single challenge. 

## Discussion

Ox-mediated CHS produces vulvar mechanical hyperalgesia, infiltration of neutrophils, upregulation of inflammatory cytokine transcripts, and increased innervation of the labiar skin in ND4 female mice. This is the first demonstration of CHS-associated hyperalgesia, and allergy-evoked *vulvar* mechanical hyperalgesia in rodents. These findings will be particularly important in elucidating the atopic mechanisms underlying vulvodynia – a chronic vulvar pain condition that is remarkably prevalent, poorly understood, and epidemiologically associated with histories of environmental allergies [8,9]. 

Mechanistic connections between allergies and pain have not been extensively studied. One study showed transient hyperalgesic responses following passive cutaneous anaphylaxis (PCA) in rats [25]. Our unpublished data indicate that pronounced thermal and mechanical hind paw hyperalgesia caused by PCA reactions in the footpad lasts up to 6 hours in mice. Active immunization and challenge with ovalbumin also caused neutrophil-dependent thermal hyperalgesia in the hind paw for up to 6 hours in rats [26]. Here, Ox-CHS provokes pronounced vulvar mechanical hyperalgesia for up to 24 hours after a single challenge and up to 5 days following 3 challenges.

Ox-sensitization has previously been shown to be mast cell dependent [3], and here we observed detectable mast cell degranulation (via reduction in tissue histamine content) in the elicitation phase 24 hours after a single Ox challenge but no difference at 24 hours after 3 Ox challenges (data not shown). We have previously shown that mast cell degranulation regulates compound 48/80-provoked short-term thermal and mechanical hyperalgesia in mice, which was abrogated by mast cell granule stabilization as well as in congenital mast cell deficiency [17]. However, here we observed that pre-treatment with sodium cromoglycate (SCG) – a compound that can act as a mast cell granule stabilizer [17] – reduced Ox-CHS hyperalgesia at 1 and 6 hours but not at 24 hours when administered 1 hour prior to Ox challenge in sensitized mice (Figure S4). It is important to note, though, that SCG can act through mast cell-independent mechanisms [27] and also does not stabilize mast cells under all conditions [28]. At this time, evidence indicates that while mast cell degranulation may contribute to CHS-induced hyperalgesia after 1 and 3 Ox challenges, it is likely not the only regulator of sustained mechanical sensitivity elicited by Ox-CHS at 24 hours. Ten or more applications of Ox have been shown to increase mast cell prevalence in the ear tissue of mice [29] and patients with provoked vulvar pain have higher than normal mast cell prevalence in their vulvar biopsy tissue [10-12]. In our ongoing studies, we are currently examining mast cell dynamics in the labiar tissue after 10 or more Ox challenges in sensitized female mice.

Labiar neutrophil influx remained elevated above baseline levels 48 hours following 1 Ox challenge by which time vulvar hyperalgesia had resolved. After 3 consecutive daily Ox challenges, neutrophil influx was upregulated at 24 hours but was indistinguishable from controls at 120 hours post-challenge when hyperalgesia was present. Though neutrophils can mediate pain responses following passive cutaneous anaphylaxis in rats [26] and mast cell degranulation in mice [17], neutrophil influx does not appear to be sufficient for Ox CHS-induced vulvar hyperalgesia in ND4 mice. As repeated Ox exposures are known to induce eosinophil recruitment into the affected tissue [30], we also assessed levels of eosinophil peroxidase (EPO) enzyme activity in the labiar tissue following 3 Ox challenges and found a ~3-fold increase of EPO in the labia of Ox challenged mice over vehicle-challenged controls at both 24 and 120 hours after the third challenge (Figure S5A). Roles of eosinophils in pain cascades are poorly defined but their known roles in tissue remodeling via induction of matrix metallo-proteinase enzymes [31] suggest that models such as ours that integrate atopy and hyperalgesia may provide opportunities to dissect contributions of eosinophils to modulation of pain sensitivity following extensive tissue remodeling. 

Inflammatory cytokines are known to mediate CHS [4,6] and inflammatory pain in rodents [19–21]. Here, we found pronounced upregulation in levels of *IL-6* and *IL-1β* mRNAs after 1 and 3 Ox challenges in sensitized mice 24 hours after challenge. *IL-6* transcripts were increased ~60-fold after a single Ox challenge and ~75-fold after 3 Ox challenges. *IL-1β* transcript levels were 15-fold and ~30-fold increased over ethanol-challenge controls following 1 and 3 Ox challenges respectively. These inflammatory cytokines are also increased in vulvar biopsies from vulvar vestibulitis patients [32] but not in vaginal lavages from patients vs. controls [33]. Genetic differences in the production of IL-1β and IL-1Ra in vulvodynia patients vs. controls [34] and enhanced IL-6 production in vulvar fibroblasts obtained from vulvodynia patients have been reported [35]. These observations further align our findings in Ox-CHS hyperalgesia with clinical evidence for a role for these inflammatory cytokines associated with allergies and vulvar pain. Recent studies of vaginal secretions in vulvodynia patients vs. controls have shown an increase in IL-4 levels in patients [33]. After 3 Ox challenges, we found a very slight upregulation in *IL-4* transcripts in the labiar tissue and are continuing to monitor the levels of this cytokine in the context of >3 Ox challenges in studies ongoing in our laboratory. Given this, and that we see detectable recruitment of eosinophils into the labiar skin after 3 Ox challenges, we assessed mRNA levels of Th2 cytokines interleukin-5 (IL-5) and interleukin-13 (IL-13) [36] and C-C motif chemokine 11/eotaxin (CCL11) 24 hours after the third Ox challenge. We found only a slight upregulation in *IL-13* mRNA levels in experimental vs. control mice, and little to no change in *IL-5* and *CCL11* transcripts (Figure S5B). Interestingly, no increases in levels of IL-5, IL-13 and CCL11 have been reported in vaginal secretions of vulvodynia patients compared to controls [33]. 

*CXCL2* transcripts showed an interesting pattern in our studies. *CXCL2* was the only cytokine gene upregulated over controls in the labia 48 hours after a single Ox challenge and was dramatically increased (~160-fold) over ethanol controls 24 hours after 3 challenges. *CXCL2* is also constitutively expressed in the brain and can play a neuro-modulatory role [37]. Therefore *CXCL2* may contribute to the lasting change in mechanical sensitivity through the modulation of innervation of allergen-challenged tissue that we see here.

Taken together, our characterization of labiar inflammation points to a sustained change in pain sensitivity after resolution of overt inflammation following 3 Ox challenges while acute inflammation and pain are present together after a single Ox challenge. Clinical vulvodynia is a chronic pain condition typically presenting without co-occurring overt inflammatory events [8]. As early as 6 hours, 3 Ox challenges provoke a smaller increase in *IL-1β* gene expression in the skin compared to a single challenge. Neutrophil influx and cytokine gene expression responses resolve by 5 days after 3 Ox challenges even as vulvar mechanical sensitivity remains detectable in challenged mice vs. controls. Therefore the model of vulvar pain we establish here reflects the reduction of acute tissue responses one would expect in chronic inflammation while preserving changed hyperalgesic sensitivity of the vulvar tissue. 

Density of CGRP^+^ and PGP 9.5^+^ cutaneous nerve fibers increased significantly in the labiar skin of Ox-challenged mice supporting earlier findings in murine ear tissue [5] and echoing similar observations in vulvodynia [11,12] and atopic dermatitis patients [38]. A slightly lower nerve density at 5 days after 3 challenges vs. 48 hours after a single challenge suggests that possible central nervous system reorganization [39] associated with repeated allergen challenge may contribute to the sustained hyper-innervation when immediate peripheral inflammatory stimuli are no longer present. Treatment with the tri-cyclic anti-depressant amitriptyline has been modestly effective in the clinical management of vulvodynia [8]; in our experiments, amitriptyline pre-treatment reduced vulvar hyperalgesia at 1, 6 and 24 hours post-challenge in mice (Figure S4). Amitriptyline abrogates neuropathic allodynia [40] and formalin pain [41] in rats and may reduce Ox CHS-pain via direct effects on nerve fibers [42] suggesting that increased cutaneous nerve density may be a key regulator of allergic vulvar hyperalgesia. 

Mast cells and nerves are known to reside in close proximity in tissues [43] mediated by adhesion molecules including N-cadherin and cell adhesion molecule 1 [44,45]. We, and others [5], show that 1-3 Ox challenges increase local nerve density, while an increase in mast cell abundance at the challenge site following 10 or more Ox challenges has been documented [29]. Furthermore, both mast cells and neurons are known to regulate each other’s functions. Proximity to neurons enhances FcεR1 expression on mast cells thus lowering the latter’s activation and degranulation thresholds [46]. In turn, mast cell-derived TNF-α and NGF can promote nerve elongation [5] and lower the threshold of nociceptor firing [47]. Therefore, in hyper-innervated allergic sites in Ox-challenged labia, mast cell-neuron synapses can potentially regulate changes in pain sensitivity in the periphery. Systemic mast cell degranulation can be followed by changes in c-fos and pERK expression in the spinal cord [48,49], indicating that mast cells may contribute to central sensitization in nociceptive signaling.

Our findings recapitulate increased inflammatory cytokine production [32], hyperinnervation of the vestibular tissue [10–12] and heightened sensitivity to mechanical pressure [50] seen in vulvodynia patients. We further corroborate recent observations [15] that repeated vulvo-vaginal infection with *Candida albicans* resulted in altered ano-genital mechanical sensitivity of mice *after* resolution of active infection. While that study did not investigate whether there was an allergic response to *C. albicans* antigens in hyperalgesic mice, our studies together provide strong evidence that even after inflammation (allergic or infection-related) is resolved, tissue innervation and pain perception can be altered in a sustained manner. We are currently adapting our model of allergen-evoked vulvar hyperalgesia to longer-term repeated allergen challenges. Parameters influencing lasting hyperalgesic responses in the vulva including the dynamics of immune cell infiltration in the tissue during and after CHS, neuro-immune interactions, changes in quality and nature of tissue cytokine production are all areas of investigation that we, and others, can build upon the foundation we present here to dissect the mechanistic relationship between allergies and hyperalgesia in mice. 

As a diagnosis of exclusion [8], vulvodynia, like many complex disorders, is likely a multivalent and multifactorial condition with diverse underlying mechanisms, including allergy, for different individuals. The synergy of epidemiological [9,51], clinical [10–12] and pre-clinical studies [15] including ours is required to dissect the complex nuances of this underserved condition, elucidate the variety of possible underlying causes and develop targeted therapies that will allow physicians and patients to manage and treat vulvodynia more successfully. Importantly, other complex pain conditions are associated with allergies or elevated mast cell functions. For example, individuals with a history of allergies are over-represented in those suffering from migraine pain [52]. Men who suffer from chronic pelvic pain syndrome are frequently co-diagnosed with chronic prostatitis and show elevated levels of mast cell mediators in prostatic secretions [53]. Therefore, the particular line of investigation we initiate here will have implications beyond vulvodynia as a tool to decipher the complex contributions of allergic reactions to chronic pain conditions.

## Supporting Information

Figure S1 **Ox challenge is applied topically onto the labia of sensitized mice.** Mice were sensitized on Day 1 with 2% Ox on the shaved flank. On Day 5, we challenged mice with 1% Ox or vehicle (100% EtOH; 40µl) using a 50µl Hamilton syringe barrel to apply either solution onto the shaved labiar skin (cross-hatched region). To quantify gene expression, cutaneous nerve density, and eosinophil peroxidase activity, we used labia (red rectangle) excised after euthanasia, while a slightly larger area (dotted circle) was used for myeloperoxidase activity assays. [Artwork: Charles Cosimini].
(TIF)

Figure S2 **Neither stage of estrous nor Ox sensitization alone alter vulvar mechanical sensitivity.** Estrous stages of mice were determined as previously described [54]. Briefly, vaginal lavages were performed using 100 µL of double-distilled H_2_0, and vaginal smears prepared by cytospin on 5 consecutive days at the same time of day (noon) immediately after von Frey measurements were taken. Vaginal smears were stained with 0.1% crystal violet and relative ratios of the predominant cell types were determined using an Olympus CKX41SF inverted binocular microscope to determine the estrous stage of each mouse. Stages of the mice’s estrous cycle (A) do not affect baseline vulvar mechanical sensitivity. Ox sensitization alone, without challenge, (B) does not produce hyperalgesia. Labiar oxazolone challenge does not alter mechanical withdrawal thresholds of the hind paws in sensitized mice (C). n = 7-12 per treatment group.
(TIF)

Figure S3 **IL-1β but not TNF-α transcripts are significantly elevated in Ox-challenged mice over controls as early as 6 hours after 1 and 3 labiar** **Ox challenges**. Sensitized mice challenged once with labiar Ox show a ~ 60-fold elevation of IL-1β mRNA levels and little difference in TNF-α transcript levels in the labiar skin compared to mice challenged with vehicle alone 6 hours after challenge (black bars). Similarly, sensitized mice challenged 3 times with Ox on the labia have 12-fold increase in *IL-1β* and little to no change in *TNF-α* mRNA compared to controls 6 hours after the third challenge (white bars).
(TIF)

Figure S4 **Amitriptyline and sodium cromoglycate pre-treatment reduce vulvar hyperalgesia in Ox-sensitized and challenged ND4 Swiss mice.** Sensitized mice pre-treated with amitriptyline (10 mg/kg ; 100µl i.p. [55]) or sodium cromoglycate (160 mg/kg; 100µl i.p.) 1 hour before Ox challenge show reduced mechanical hyperalgesia at 1, 6, and 24h after Ox challenge. Significances are compared to Ox/Sal/Ox (* = p<0.05,** = p<0.01, *** = p<0.0001). n=10 mice per treatment group; data represent 2-3 independent experiments.
(TIF)

Figure S5 **Three consecutive labiar oxazolone challenges induce an increase in eosinophil activity in the labiar tissue of female ND4 Swiss mice.** Mice that received 3 labiar Ox challenges following sensitization showed an increase in eosinophil peroxidase activity in the labiar tissue at 24 hours and 120 hours (A); to measure eosinophil peroxidase activity (EPO), we adapted previously described methods [56,57]; excised labia were preserved at -80°C in 0.5% hexadecyltrimethyl ammonium bromide in phosphate-buffered saline, homogenized, freeze-thawed three times, centrifuged twice, first at 4750 rpm and next at 14000 rpm for 4 minutes at 4°C, and 50µl of the supernatant mixed with 100µl of 16mM o-phenylenediamene, 50mM-Tris-HCL, 0.01% H_2_O_2_. The reaction was stopped with 1M H_2_SO_4_ after 15 minutes and OD measurements taken at 490 nm. EPO activity was represented as OD/g of wet tissue. At 24 hours after 3 Ox challenges, mice showed a slight upregulation of *IL-13* (Mm00434204_m1) mRNAs in the labiar skin but there was little to no change in *eotaxin/CCL11* (Mm00441238_m1) and *IL-5* (Mm00439646_m1) transcript levels by qRT-PCR analysis (B). Significances are compared to Ox/EtOH (* = p<0.05, ** = p<0.01, *** = p< 0.0001 respectively). n = 2-6 mice per treatment group; data represent at least 2 independent experiments.
(TIF)

Table S1 **Average raw cycle threshold (Ct) values showing detectable *IL-4* mRNA (Mm00445259_m1) transcripts at 24 hours following triple Ox challenge in sensitized mice.**
(DOCX)
