# Supplementary figures and images for: Contact Hypersensitivity to Oxazolone Provokes Vulvar Mechanical Hyperalgesia in Mice
Source: PLoS One. 2013 Oct 25;8(10):e78673. doi: 10.1371/journal.pone.0078673 (PMC3808293; doi:10.1371/journal.pone.0078673)

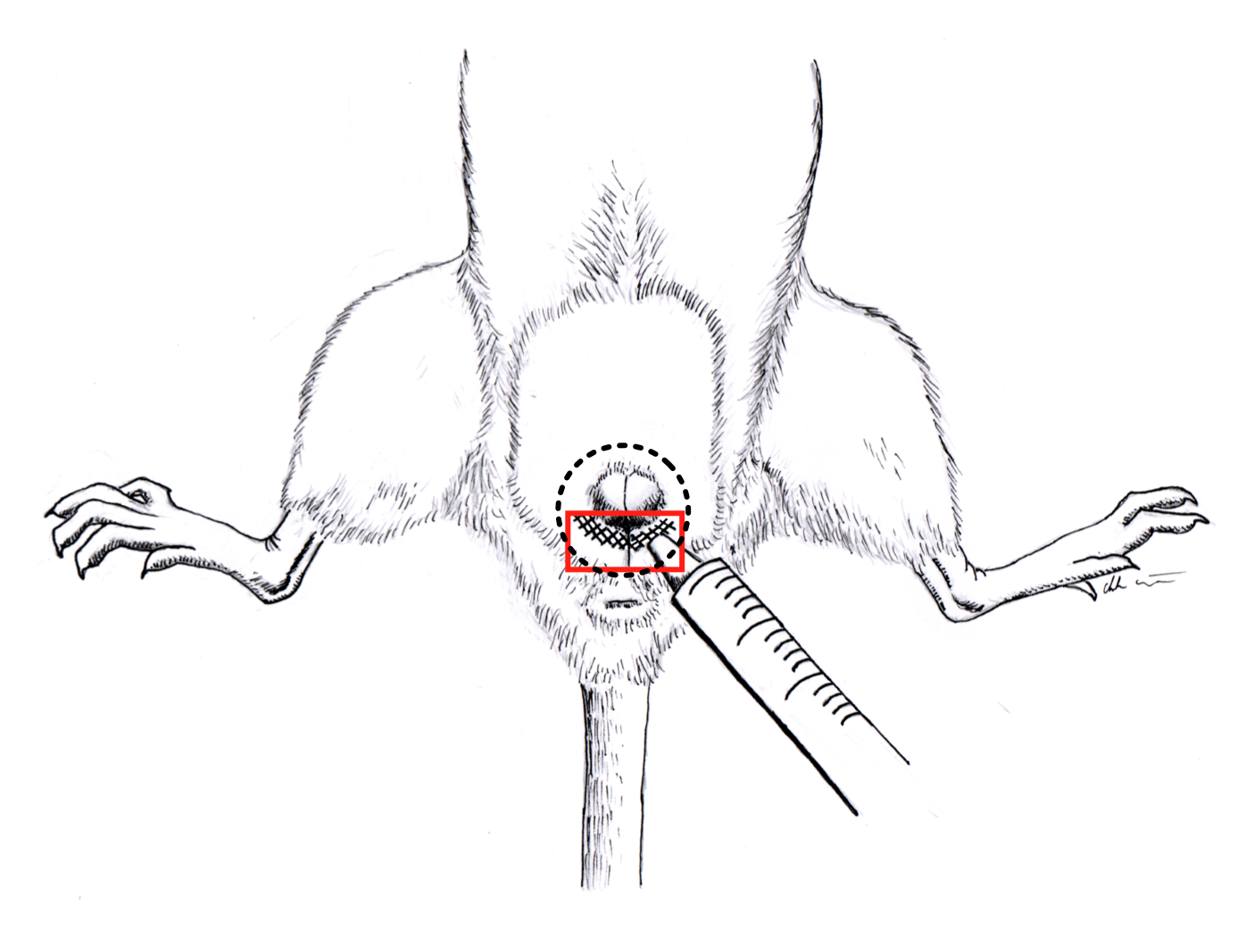

Supplement: Figure S1 — Ox challenge is applied topically onto the labia of sensitized mice. Mice were sensitized on Day 1 with 2% Ox on the shaved flank. On Day 5, we challenged mice with 1% Ox or vehicle (100% EtOH; 40µl) using a 50µl Hamilton syringe barrel to apply either solution onto the shaved labiar skin (cross-hatched region). To quantify gene expression, cutaneous nerve density, and eosinophil peroxidase activity, we used labia (red rectangle) excised after euthanasia, while a slightly larger area (dotted circle) was used for myeloperoxidase activity assays. [Artwork: Charles Cosimini]. (TIF) [file pone.0078673.s001.tif]

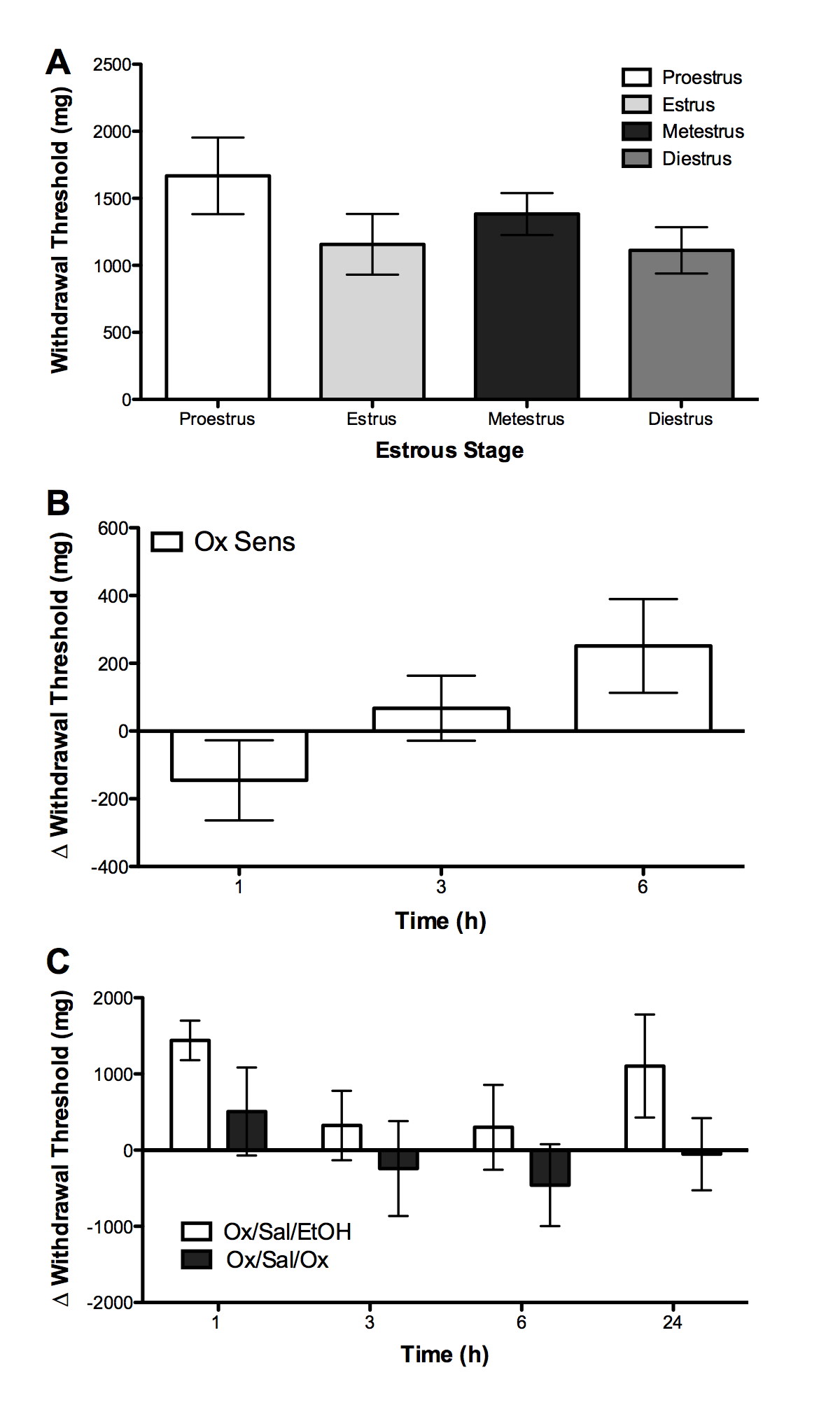

Supplement: Figure S2 — Neither stage of estrous nor Ox sensitization alone alter vulvar mechanical sensitivity. Estrous stages of mice were determined as previously described [54]. Briefly, vaginal lavages were performed using 100 µL of double-distilled H20, and vaginal smears prepared by cytospin on 5 consecutive days at the same time of day (noon) immediately after von Frey measurements were taken. Vaginal smears were stained with 0.1% crystal violet and relative ratios of the predominant cell types were determined using an Olympus CKX41SF inverted binocular microscope to determine the estrous stage of each mouse. Stages of the mice’s estrous cycle (A) do not affect baseline vulvar mechanical sensitivity. Ox sensitization alone, without challenge, (B) does not produce hyperalgesia. Labiar oxazolone challenge does not alter mechanical withdrawal thresholds of the hind paws in sensitized mice (C). n = 7-12 per treatment group. (TIF) [file pone.0078673.s002.tif]

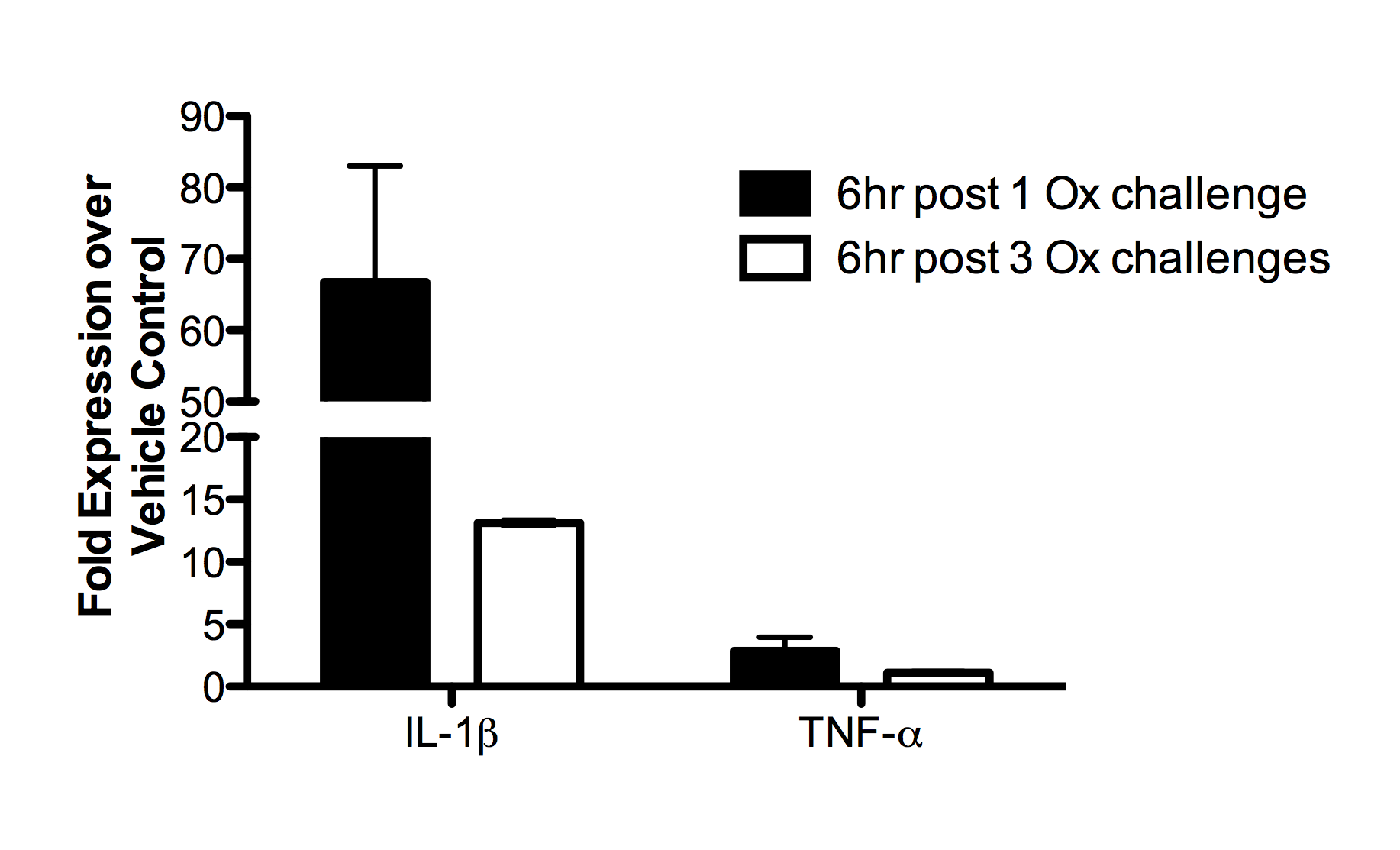

Supplement: Figure S3 — IL-1β but not TNF-α transcripts are significantly elevated in Ox-challenged mice over controls as early as 6 hours after 1 and 3 labiar Ox challenges. Sensitized mice challenged once with labiar Ox show a ~ 60-fold elevation of IL-1β mRNA levels and little difference in TNF-α transcript levels in the labiar skin compared to mice challenged with vehicle alone 6 hours after challenge (black bars). Similarly, sensitized mice challenged 3 times with Ox on the labia have 12-fold increase in IL-1β and little to no change in TNF-α mRNA compared to controls 6 hours after the third challenge (white bars). (TIF) [file pone.0078673.s003.tif]

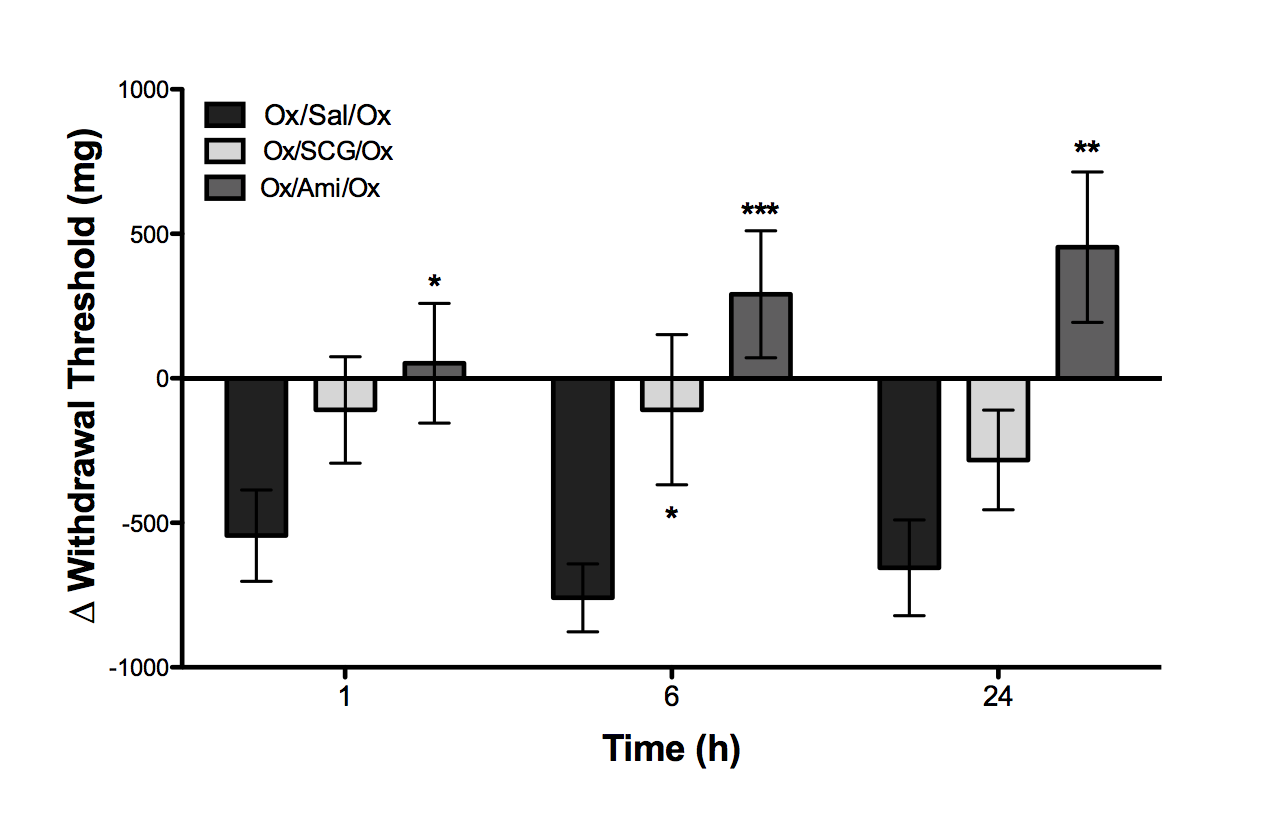

Supplement: Figure S4 — Amitriptyline and sodium cromoglycate pre-treatment reduce vulvar hyperalgesia in Ox-sensitized and challenged ND4 Swiss mice. Sensitized mice pre-treated with amitriptyline (10 mg/kg ; 100µl i.p. [55]) or sodium cromoglycate (160 mg/kg; 100µl i.p.) 1 hour before Ox challenge show reduced mechanical hyperalgesia at 1, 6, and 24h after Ox challenge. Significances are compared to Ox/Sal/Ox (* = p<0.05,** = p<0.01, *** = p<0.0001). n=10 mice per treatment group; data represent 2-3 independent experiments. (TIF) [file pone.0078673.s004.tif]

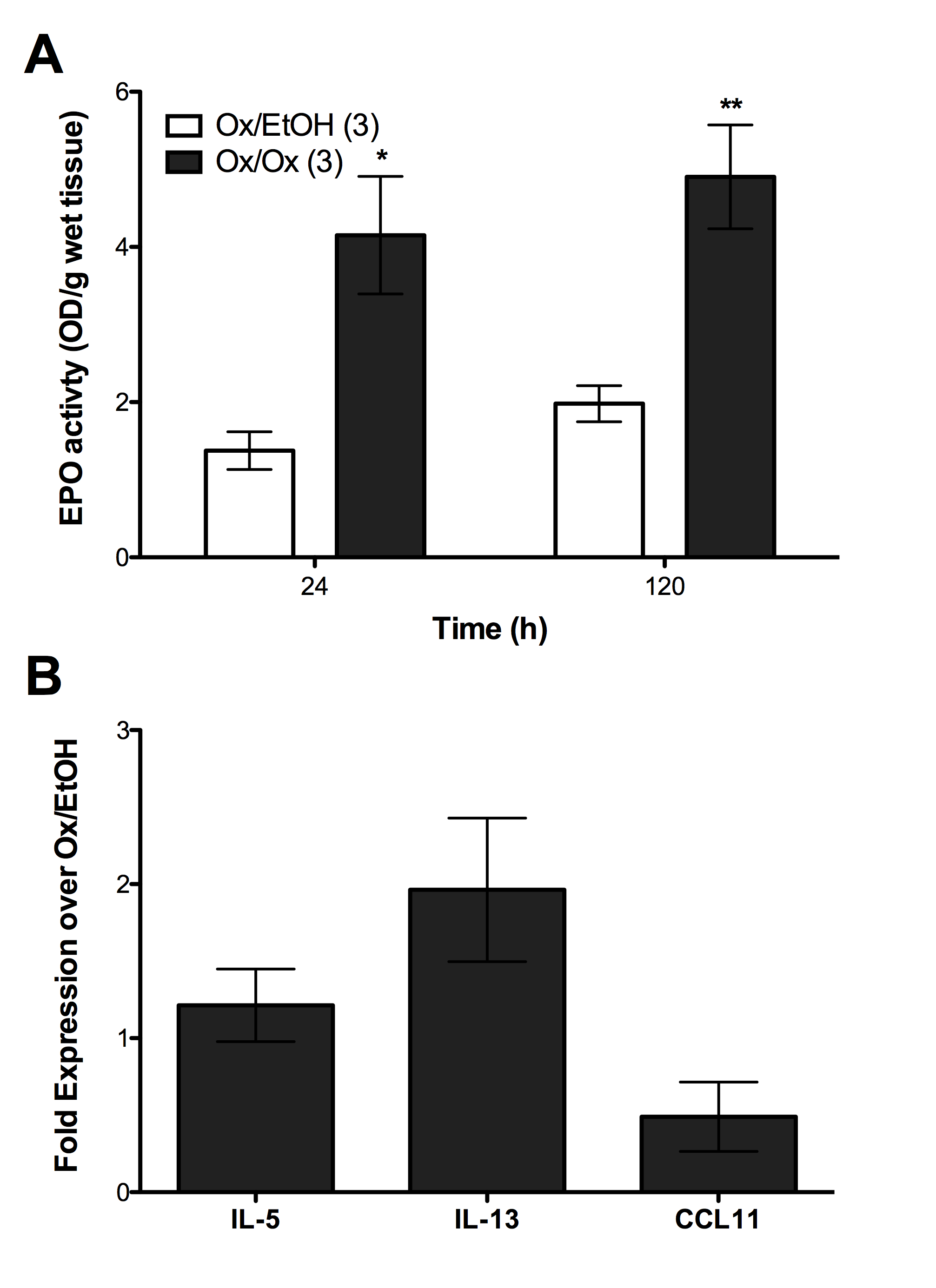

Supplement: Figure S5 — Three consecutive labiar oxazolone challenges induce an increase in eosinophil activity in the labiar tissue of female ND4 Swiss mice. Mice that received 3 labiar Ox challenges following sensitization showed an increase in eosinophil peroxidase activity in the labiar tissue at 24 hours and 120 hours (A); to measure eosinophil peroxidase activity (EPO), we adapted previously described methods [56,57]; excised labia were preserved at -80°C in 0.5% hexadecyltrimethyl ammonium bromide in phosphate-buffered saline, homogenized, freeze-thawed three times, centrifuged twice, first at 4750 rpm and next at 14000 rpm for 4 minutes at 4°C, and 50µl of the supernatant mixed with 100µl of 16mM o-phenylenediamene, 50mM-Tris-HCL, 0.01% H2O2. The reaction was stopped with 1M H2SO4 after 15 minutes and OD measurements taken at 490 nm. EPO activity was represented as OD/g of wet tissue. At 24 hours after 3 Ox challenges, mice showed a slight upregulation of IL-13 (Mm00434204_m1) mRNAs in the labiar skin but there was little to no change in eotaxin/CCL11 (Mm00441238_m1) and IL-5 (Mm00439646_m1) transcript levels by qRT-PCR analysis (B). Significances are compared to Ox/EtOH (* = p<0.05, ** = p<0.01, *** = p< 0.0001 respectively). n = 2-6 mice per treatment group; data represent at least 2 independent experiments. (TIF) [file pone.0078673.s005.tif]
