# Supplementary material for: Contact Hypersensitivity to Oxazolone Provokes Vulvar Mechanical Hyperalgesia in Mice
Source: PLoS One. 2013 Oct 25;8(10):e78673. doi: 10.1371/journal.pone.0078673 (PMC3808293; doi:10.1371/journal.pone.0078673)
Supplement: Table S1 — Average raw cycle threshold (Ct) values showing detectable IL-4 mRNA (Mm00445259_m1) transcripts at 24 hours following triple Ox challenge in sensitized mice. (DOCX) [file pone.0078673.s006.docx]

**Table S1.Average raw cycle threshold (Ct) values showing detectable *IL-4* mRNA (Mm00445259_m1) transcripts at 24 hours following triple Ox challenge in sensitized mice.**

|  | **NT** | **Ox/EtOH (3)** | **Ox/Ox (3)** |
| --- | --- | --- | --- |
| **Ct value (*β2m*)** | 21.53 | 21.36 | 19.76 |
| **Ct value (*IL-4*)** | N.D. | N.D. | 32.13 |
